# Neuronal Hyperactivation in EEG Data during Cognitive Tasks Is Related to the Apolipoprotein J/Clusterin Genotype in Nondemented Adults

**DOI:** 10.3390/ijms24076790

**Published:** 2023-04-05

**Authors:** Natalya V. Ponomareva, Tatiana V. Andreeva, Maria S. Protasova, Svetlana S. Kunizheva, Irina L. Kuznetsova, Ekaterina P. Kolesnikova, Daria D. Malina, Andrey A. Mitrofanov, Vitaly F. Fokin, Sergey N. Illarioshkin, Evgeny I. Rogaev

**Affiliations:** 1 Research Center of Neurology, 125367 Moscow, Russia; 2 Center for Genetics and Life Science, Sirius University of Science and Technology, 354349 Sochi, Russia; 3 Vavilov Institute of General Genetics, Russian Academy of Sciences, 119991 Moscow, Russia; 4 Centre for Genetics and Genetic Technologies, Faculty of Biology, Lomonosov Moscow State University, 119192 Moscow, Russia; 5 Research Center of Mental Health, 115522 Moscow, Russia; 6 Department of Psychiatry, Umass Chan Medical School, Shrewsbury, MA 01545, USA

**Keywords:** Alzheimer’s disease, genetic predisposition, *CLU* genotype, EEG, event-related-desynchronization, verbal fluency, aging

## Abstract

The clusterin (*CLU*) rs11136000 *CC* genotype is a probable risk factor for Alzheimer’s disease (AD). *CLU*, also known as the apolipoprotein *J* gene, shares certain properties with the apolipoprotein E (*APOE*) gene with a well-established relationship with AD. This study aimed to determine whether the electrophysiological patterns of brain activation during the letter fluency task (LFT) depend on *CLU* genotypes in adults without dementia. Previous studies have shown that LFT performance involves activation of the frontal cortex. We examined EEG alpha1 and alpha2 band desynchronization in the frontal regions during the LFT in 94 nondemented individuals stratified by *CLU* (rs11136000) genotype. Starting at 30 years of age, *CLU CC* carriers exhibited more pronounced task-related alpha2 desynchronization than *CLU CT&TT* carriers in the absence of any differences in LFT performance. In *CLU CC* carriers, alpha2 desynchronization was significantly correlated with age. Increased task-related activation in individuals at genetic risk for AD may reflect greater “effort” to perform the task and/or neuronal hyperexcitability. The results show that the *CLU* genotype is associated with neuronal hyperactivation in the frontal cortex during cognitive tasks performances in nondemented individuals, suggesting systematic vulnerability of LFT related cognitive networks in people carrying unfavorable *CLU* alleles.

## 1. Introduction

Alzheimer’s disease (AD), is a progressive and irreversible neurodegenerative disease which leads to cognitive decline and memory impairment. AD is the most common form of dementia, affecting approximately 50 million people worldwide [1]. The major histopathological hallmarks of AD include extracellular amyloid plaques and intracellular neurofibrillary tangles, composed of abnormally hyperphosphorylated tau protein [2]. Age is the most significant risk factor for AD, with the incidence of the disease increasing exponentially after the age of 65 [3].

AD is strongly linked to genetics, with heritability estimates of 59–79% [4]. Mutations in three genes have been identified as causative factors for familial AD, including the amyloid precursor protein (*APP*) gene (chr 21), the presenilin 1 (*PSEN1*) gene (chr14), and presenilin 2 (*PSEN2*) (chr1) [4,5,6,7,8]. The most common genetic risk factor for AD in Caucasian groups, including the Russian population, is a polymorphism in apolipoprotein E (*APOE*) (chr19) [9,10,11,12,13].

GWAS have provided evidence of AD risk genes: clusterin *CLU* (chr8) and *PICALM* (chr11) [14,15]. The major SNP influencing the risk of AD is rs11136000, which is located in intron 3 of the *CLU* gene [16,17]. *CLU* is now considered the third greatest risk gene for late onset Alzheimer’s disease (LOAD), after *APOE* and *BIN1* [18]. The risk of developing LOAD is 1.16 times higher with the presence of the *CLU C* allele than the T allele [18]. *CLU* rs11136000 polymorphisms have also been linked to an increased risk of MCI [19], as well as the progression from MCI to AD [20]. *CLU*, also known as apolipoprotein J, has a number of properties similar to *APOE*. *CLU* is abundantly expressed in the brain [21]. *CLU* and *APOE* act as amyloid-beta (Aβ) chaperones that can alter Aβ aggregation and/or clearance [18,22]. In AD, *CLU* is found to be upregulated in the hippocampus and cortex, where it colocalizes with amyloid beta (Aβ) plaques. Additionally, *CLU* is upregulated in the cerebrospinal fluid (CSF) of AD patients. The interaction between *CLU* and Aβ appears to alter Aβ aggregation and promote Aβ clearance, indicating a potential neuroprotective function [18]. However, other studies have shown that *CLU* reduces Aβ clearance and increases Aβ-induced neurotoxicity; thus, the nature of the interaction may depend on the ratio of *CLU* to Aβ [23]. *CLU* and *APOE* are involved in the transport of cholesterol and phospholipids [21,24,25]. The influence of membrane-bound cholesterol on various transmembrane receptors and enzymes, such as β-secretase and γ-secretase which produce Aβ by cleaving amyloid precursor protein, suggests that *CLU* may alter the risk of LOAD by regulating cholesterol metabolism. *CLU* modulates AD-related pathways such as those of inflammation and apoptosis [18].

Early identification of functional brain changes associated with genetic susceptibility to AD is essential for elucidating the pathological processes and, potentially, developing pharmacological interventions. The definition of AD has recently been updated to include the preclinical stage, which is characterized by the presence of at least one biomarker of AD pathology, but no symptoms of cognitive impairment. These biomarkers include decreased levels of Aβ42, increased levels of t-tau and p-tau in cerebrospinal fluid, or abnormal deposition of Aβ and tau in the brain as detected by positron emission tomography [26]. Determining the effects of AD risk gene variants on the brain would help to characterize the mechanisms of those risk alleles, enabling the development of more targeted therapeutic treatments.

Quantitative EEG (QEEG) is a valuable diagnostic tool in the study of dementia [27,28,29,30,31,32,33,34]. Brain oscillations represent the basic mechanisms of neural communication and reflect the processes of excitation and inhibition in neuronal networks. The heritability of EEG patterns has been estimated to be between 70% and 90% [35]. EEG and MEG can be used to identify endophenotypes, which are the basic heritable quantitative biological markers that can be detected even at the preclinical stage of the disease.

EEG may have a predictive value for future AD development in normal subjects [36,37].

Research has demonstrated an association between QEEG parameters and AD risk variants in the *APOE*, *CLU*, *PICALM*, *IL1RAP*, *UNC5C*, and *NAV2* genes in AD [38,39,40,41,42] and MCI patients [43,44,45] as well as in healthy adults [46,47,48,49,50]. Moreover, the association of neurophysiological parameters with AD-related genetic factors was shown to be age dependent. In an MEG study, increased neural activity (specifically in the theta band) during a working visual memory task was found in cognitively intact young *APOE E4* carriers when compared with *APOE E4* noncarriers [51].

EEG variables, particular alpha band event-related desynchronization/synchronization (ERD/S), characterize important processes underlying human cognition [52,53]. ERD reflects brain activation during cognitive tasks and correlates with fMRI BOLD responses, which have been shown to differ depending on *CLU* and *APOE* genotypes [54,55].

The effect of *CLU* genotypes on cognitive task-related alpha band ERD/S has not been previously investigated.

Covert word retrieval was the cognitive task used in this study, as tested with the letter fluency test (LFT). The LFT has been used in studies of ERD/S. Regions with LFT–related desynchronization of brain oscillations in the 5–15 Hz frequency ranges, as detected using MEG, align with areas of the brain showing BOLD response on fMRI [56]. LFT is one of the most commonly used versions of the Controlled Oral Word Association Test (COWAT) [57].

Verbal fluency is a cognitive function that requires executive and language abilities. Previous neuroimaging studies have shown that LFT performance is mediated primarily by the frontal cortex. Peak activations during phonemic verbal fluency are mainly located in the left inferior/middle frontal gyrus and the anterior cingulate gyrus [58]. Additionally, left frontal lesions were more detrimental to phonemic fluency than right frontal lesions [59].

The effect of *CLU* genotypes on LFT-related alpha ERD/S has not been previously investigated.

The study aimed to determine whether EEG patterns of brain activation, in particular, LFT-related alpha band ERD/S in frontal regions in nondemented adults, depend on the *CLU* rs11136000 genotype, and whether this effect is modified by aging.

## 2. Results

There were no significant differences in age, *APOE* genotype, or LFT performance between *CLU CC* and the *CLU CT&TT* carriers (Table 1). The proportion of men and women differed in the groups with *CLU CC* and *CLU CT&TT* genotypes.

**Figure 1:**
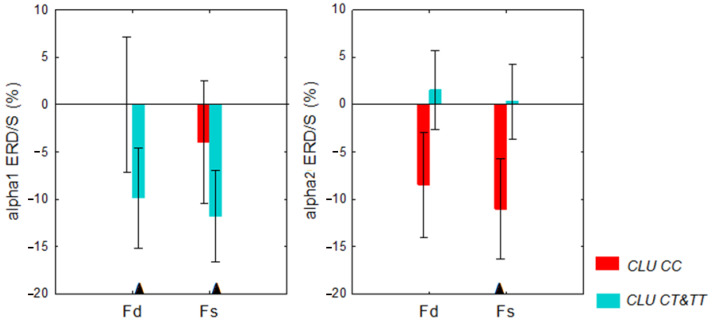
Alpha1 and alpha2 desynchronization/synchronization (ERD/S) in the right (Fd) and left (Fs) frontal regions during the letter fluency test (LFT) in nondemented individuals aged 19 to 80 years with different *CLU* genotypes. Triangle indicates significant ERD/S.

In *CLU CT&TT* individuals, the LFT elicited alpha1 ERD in Fd (*p* = 0.04) and Fs regions (*p* = 0.02), while alpha2 ERD was not significant. In the *CLU CC* carriers, LFT-induced alpha2 ERD was significant in Fs regions (*p* = 0.02), but not significant in Fd regions. In *CLU CC* carriers, the changes in alpha1 power during the LFT were not significant (Figure 1). The effect of sex on the ERD/S during the LFT was not significant (*p* = 0.85).

The effect of *CLU* genotype on alpha band desynchronization during LFT was significant beginning at 30 years of age. The demographic characteristics of participants aged 30 to 80 years are presented in Table 2. There were no significant differences in age, sex, *APOE* genotype, or LFT performance between the *CLU CC* and *CLU CT&TT* carriers (*p* > 0.05).

**Figure 2:**
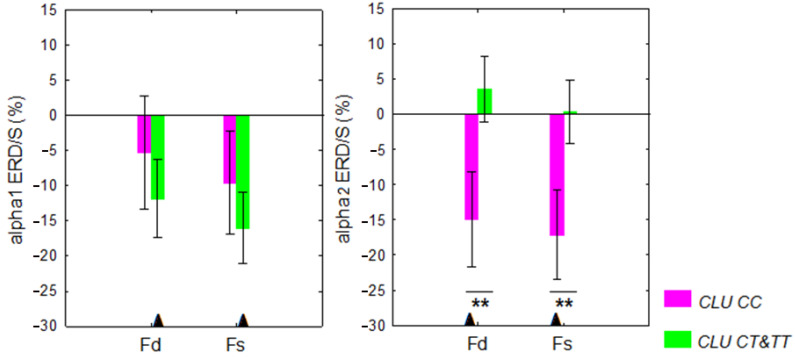
Alpha1 and alpha2 desynchronization/synchronization (ERD/S) in the right (Fd) and left (Fs) frontal regions elicited by the letter fluency test (LFT) in individuals aged 30 to 80 years with different *CLU* genotypes. ** *p* < 0.01, significant differences in ERD/S between *CLU CC* and *CLU CT&TT* carriers according ANOVA results. Abbreviations are the same as in Figure 1 and Table 1.

In *CLU CC* carriers older than 29 years of age, the LFT elicited significant alpha2 ERD in Fd (*p* = 0.002) and Fs (*p* = 0.0006) regions, whereas alpha1 ERD was not significant. In *CLU CT&TT* carriers older than 29 years of age, LFT-induced alpha1 ERD was significant in Fd (*p* = 0.022) and Fs (*p* = 0.002) regions, whereas alpha2 ERD was not significant (Figure 2). In *CLU CT&TT* carriers older than 29 years of age, the ERD difference between the left (Fs) and the right (Fd) frontal regions was significant (*p* < 0.05).

The ANOVA results revealed a main effect of the *CLU* genotype (*CLU CC* vs. *CLU CT&TT*) on alpha2 ERD/ERS F (1,67) = 5.31, *p* = 0.024). Post hoc comparison showed that the alpha2 ERD for the Fd and Fs regions was stronger in *CLU CC* genotype carriers than in *CLU CT&TT* carriers (*p* = 0.022 and *p* = 0.043 for Fd and Fs regions, respectively) (Figure 2).

The control analysis showed that the effect of the *APOE* genotype on ERD/S in the subjects aged 30 to 80 years was not significant (F = 1.76, *p* = 0.19). We detected no significant interaction between the *CLU* and *APOE* genotypes (*p* = 0.782). Thus, the effect of the *CLU* genotype on ERD was not mediated by the *APOE* genotype.

**Figure 3:**
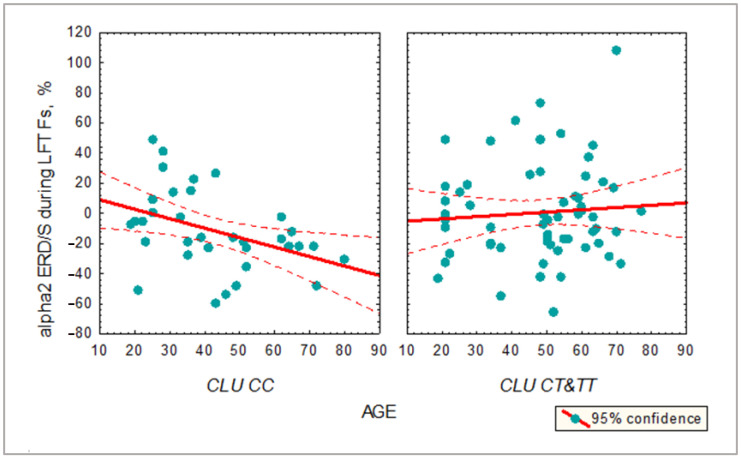
Correlation between age and alpha2 desynchronization during LFT in subjects with *CLU CC* and *CLU CT&TT* genotypes.

In the entire sample, alpha2 ERD/S values showed a significant negative correlation with age in *CLU CC* carriers only (r = −0.49, *p* = 0.012; r = −0.44, *p* = 0.023 for Fs and Fd regions, respectively), indicating higher desynchronization in older subjects with the *CLU CC* genotype during the LFT. There was no significant correlation between alpha1 ERD and age in any of the *CLU* genotype groups (Figure 3).

## 3. Discussion

The main results of this study were that in all examined groups, the LFT elicited alpha1 and alpha2 desynchronization in the frontal cortex, particularly in the left hemisphere. In *CLU CC* carriers, alpha2 ERD showed a significant correlation with age, whereas in *CLU CT&TT* carriers, alpha2 ERD was age-independent. Starting at 30 years of age, in the nondemented carriers of the AD risk variant *CLU CC*, LFT-related alpha2-desynchronization was more pronounced than in the noncarriers of this variant in the absence of any difference in the verbal fluency performance. The control analysis showed that the effect of the *APOE* genotype on alpha band ERD/S during the LFT in individuals aged 30 to 80 years was not significant and that the effect of the *CLU* genotype on alpha band ERD was not mediated by the *APOE* genotype.

The importance of alpha desynchronization in processing information has been demonstrated using a variety of different sensory-motor and cognitive paradigms [60,61,62]. Alpha ERD involves top-down network interactions [62]. Our results of desynchronization, predominantly in the left hemisphere during the LFT, are consistent with previous studies that reported higher ERD in the left hemisphere during verbal tasks [53] and, in particular, the LFT [56]. The study of covert LFT-related brain activation was assessed using MEG and fMRI data [56]. fMRI blood-oxygen-level-dependent (BOLD) response reflects changes in blood oxygenation driven by changes in local cerebral blood flow coupled to neuronal activity [63,64]. LFT-related BOLD responses were detected predominantly within the left hemisphere, including the left frontal cortex. Areas with LFT-related ERD in the 5–10 Hz and 15–25 Hz frequency ranges matched the areas with the observed BOLD response. This overlap of locations active during LFT confirmed that event-related desynchronization increased neural activation in a cortical area [52].

A greater decrease in alpha2 (11–13 Hz) power in the *CLU* CC subjects during the task implied increased task-related brain activation in subjects genetically predisposed to AD. Decreased alpha was shown to be related to the difficulty of the task, indicating that this signal is inversely related to the amount of cortical resources allocated to task performance [65]. Patients with dementia demonstrated upregulation of the bilateral frontal cortex compared to cognitively healthy older individuals when performing verb fluency tasks [66]. Increased activity of the frontal areas in patients may suggest that additional effort is required to support attention and executive functions to maintain verbal fluency. The upper alpha band may be modulated mainly by stimulus-related aspects of semantic memory processes, and the low alpha may be modulated as a function of attentional demands [67]. fMRI studies also reported hyperactivation during working memory tasks in asymptomatic *APOE E4+* and *CLU C* allele carriers [54,55]. 

A detailed analysis of the association between *APOE* genotype and alpha band ERD/S during the LFT in individuals of different ages is beyond the scope of this present study. Further research with a larger sample size is needed to determine if *APOE* genotypes have a significant effect on alpha band ERD in individuals of different ages.

Similar hyperactivation was revealed using fMRI in 18- to 26-year-old *PSEN1* E280A mutation carriers with increased CSF and plasma levels of Aβ1-42 [68]. Hyperactivation during cognitive tasks in carriers of various AD-related genotypes indicates that the phenomenon reflects processes that are important in AD pathogenesis. However, AD-related genetic factors can influence different pathogenetic pathways, potentially modifying the age at which neurophysiological alterations and cognitive decline occur. 

The ERD alterations in *CLU CC* carriers may represent early signs of AD pathology in healthy individuals. Further studies are needed to determine whether ERD alterations are related to the preclinical stage of AD or to factors contributing to the development of the disease. Studies have demonstrated that among nondemented individuals, biomarker abnormalities linked to both amyloid-dependent and amyloid-independent pathological profiles increase with aging [69].

Hyperactivation may be due to the greater cognitive effort needed to perform the task [54,55]. Hyperactivation can also result from neuronal hyperexcitability, as has been shown in *APOE E4+* carriers under hyperventilation [46]. A number of factors connected with the *CLU* genotype may cause hyperexcitability. Clusterin critically modifies Aβ clearance from the brain across the blood–brain barrier [70] and alters Aβ aggregation [21]. Recent experimental evidence implies that at intermediate levels of Aβ pathology, presynaptic facilitation leads to synaptic potentiation and excitation, whereas higher levels of Aβ induce postsynaptic depression [71].

Our previous study showed that older *CLU CC* carriers have higher alpha3 power in resting-state EEG than *CLU CT&TT* carriers [48]. Previous studies on the interrelation between resting-state alpha activity and ERD have reported that alpha synchronization in spontaneous EEG is associated with ERD when performing different tasks [52,72]. Thus, higher spontaneous upper alpha activity in *CLU CC* carriers compared to noncarriers may drive ERD during the LFT.

Individuals at higher genetic risk for AD may exhibit hypersynchrony and abnormal activation of cortical and hippocampal networks due to the loss of tonic inhibition [46,48,49,73]. Inhibitory deficits in these networks may impair subsequent learning and memory [71,73].

fMRI studies have demonstrated an association of hippocampal hyperactivity with cortical thinning, specifically in the posteromedial and lateral temporoparietal cortices [74]. The authors proposed that hippocampal hyperactivity might represent neuronal excitotoxicity with impending synaptic failure and incipient cognitive decline [75].

One study [76] showed a correlation between the *CLU* risk variant rs11136000 and decreased integrity of widespread white matter regions in healthy young adults, as revealed through diffusion tensor imaging. The *CLU* rs11136000 *C* allele predicts increased ventricular expansion in the brain, regardless of dementia status or *APOE* genotype [77]. The study also indicated that the *CLU* and *APOE* risk variants have a combined effect on both volumetric enlargement and the shape of the lateral ventricles. An fMRI study also indicated abnormal activation of the dorsolateral prefrontal cortex and hippocampus during working memory tasks in healthy young individuals with the *CLU* rs11136000 risk variant *C* [55,78]. 

These convergent lines of evidence imply that hyperactivation during cognitive tasks in *CLU CC* carriers might be due to increased vulnerability of their LFT-related cognitive brain networks, probably related to the neurotoxicity of small amounts of Aβ. These EEG features provide valuable insights into the neural mechanisms underlying the relationship between genetic influences and AD development.

## 4. Materials and Methods

### 4.1. Participants

The enrolled cohort included 94 nondemented individuals, 38 men and 56 women, age range 19–80 years. All subjects were Russian and were from Moscow and the greater Moscow region. Subjects underwent neurological examination and cognitive screening. The included subjects were free of dementia and other medical, psychiatric, and neurological conditions. The exclusion criteria were as follow: a personal history of mental illness, signs of anxiety or depression, physical brain damage, neurological disorder or other medical condition, e.g., hypertension, diabetes, cardiac disease, and thyroid disease, or a personal history of drug or alcohol use disorder. Subjects were evaluated with the MMSE and Clinical Dementia Rating (CDR) scale [79], and only subjects with MMSE scores of 28 and more and a CDR score of 0 were included in the study. All subjects were right-handed.

Informed written consent was obtained from all participants included in the study. The experimental protocol of this study was approved by the local ethics committee.

Subjects were divided into subgroups according to their *CLU* genotypes (*CLU CC* and *CLUCT&TT).*

### 4.2. Procedure

During the experiments, the subjects sat comfortably in a chair. They were asked to close their eyes and relax during the recording. The technician continuously monitored the subjects’ vigilance by evaluating the EEG and observing the subjects. The first phase involved 3 min of rest.

The task condition encompassed the letter fluency test, which consisted of a single 6 min session divided into 6 epochs, each 60 s in length. The first epoch was an active silent *epoch* in which subjects were instructed to think of as many words as possible beginning with the given letter. The second epoch was an active aloud epoch in which subjects spoke aloud the invented words and other words starting with the given letter. This silent/spoken design was repeated 3 times with three different letters. The technician spoke each of the three letters at the beginning of the corresponding active silent epoch.

Prior to data acquisition, the participants received approximately 8 min of training. After one or two training sessions, all participants reported that they fully understood the instructions.

### 4.3. EEG Recording

EEG evaluation and recording was carried out in accordance with the International Pharmaco-EEG Society (IPEG) guidelines [80]. EEGs were recorded during resting and during 6 min of the LFT. A Nihon Kohden 4217 G EEG device was used, with a time constant of 0.3 s and a high-frequency cutoff of 45 Hz. The 16 Ag/AgCl electrodes were placed according to the international 10–20 system at the O2, O1, P4, P3, C4, C3, F4, F3, FP2, FP1, T6, T5, T4, T3, F8, and F7 positions, with linked ears serving as the reference. Electrode impedance did not exceed 10 κΩ. EEG data were simultaneously sampled at 256 Hz per channel from 180 s of resting and 180 s of active silent (LFT) conditions and stored on a computer for off-line analysis. Visual inspection of the data was then conducted to eliminate any periods of artifacts. After removing artifacts, the EEG data in frontal regions (F3, F4, Fp2, Fp1) during the LFT (active silent) and resting periods were selected for further analysis.

### 4.4. Genetic Analysis

Genomic DNA was isolated from peripheral venous blood samples by using a QIAamp DNA Blood Midi Kit (Qiagen, Hilden, Germany). Genotyping was performed using PCR, followed by a restriction fragment length polymorphism (RFLP) analysis. Amplification was performed according to the manufacturer’s instructions using both the GeneAmp PCR System 9700 thermal cycler (Applied Biosystems, Thermo Fisher Scientific, Waltham, MA, USA) and the Veriti™ 96-Well Fast Thermal Cycler (Thermo Fisher Scientific, Waltham, MA, USA).

To genotype the *APOE* gene locus, the following oligonucleotide primers were used: CGGCTGGGCGCGGACATGGAGGA and TCGCGGGCCCCGGCCTGGTACAC. The PCR protocol was as follows: preliminary denaturation at 95 °C for 4 min; 5 cycles: 95 °C for 45 s, 64 °C for 25 s, and 72 °C for 30 s; and 30 cycles: 95 °C for 5 s, 64 °C for 15 s, and 72 °C for 5 s, followed by a final stage at 72 °C for 3 min. PCR products were then cleaved using *BstHH*I (SibEnzyme, Novosibirsk, Russia) and restriction products were analyzed in 7.5% polyacrylamide gels.

The *rs11136000* polymorphism in the *CLU* gene was determined with PCR using the following oligonucleotide primers: 5′_CTTTGTAATGATGTACCATCTACCC and 3′_AGGCTGCAGACTCCCTGAAT. The PCR protocol was as follows: preliminary denaturation at 95 °C for 1 min and 35 cycles of 94 °C for 30 s, 57 °C for 30 s, and 72 °C for 1 min. The last stage was performed at 72 °C. The 645 bp PCR products were then cleaved by *Acs*I restriction endonuclease (SibEnzyme, Novosibirsk, Russia) and restriction fragments were analyzed in a 2% agarose gel.

### 4.5. Data Analysis

EEG data were collected under two different conditions: resting with eyes closed and silent LFT with the eyes closed.

A total of 35–40 4 s epochs free of artifacts of resting and task-based EEG were processed by fast Fourier transform. The obtained spectra were averaged to obtain a mean power spectrum for each channel.

For the EEG spectral analysis, the frequency bands of interest were alpha1 (8–10.99) and alpha2 (11–12.99). The event-related desynchronization/synchronization (ERD/ERS) of alpha1 and alpha2 was calculated as follows:

ERD% = 100 × (E − R)/R(1)

where E indicates the absolute power at the “event” LFT and R is the absolute power at “rest”.

The resting absolute powers and ERD/S during the LFT were calculated in the cortical regions of interest as follows: frontal right Fd = (F4 + Fp2)/2 and left Fs = (F3 + Fp1)/2.

The EEG parameters from the groups were assessed for normality using the Shapiro–Wilk test, and the results showed no evidence of skewness in the data. General linear models (GLM) and post hoc Duncan tests with a significance threshold of *p* = 0.05 were used to compare EEG parameters (alpha1 and alpha2 ERD) between the groups with different *CLU* genotypes (*CLU CC* vs. *CLU CT&TT*) with control variables *APOE*, age, and sex included as covariates or fixed factors as appropriate.

We performed Pearson’s correlations of age with alpha1 ERD/S, alpha2 ERD/S, and LFT performance.

## 5. Conclusions

This study was the first to demonstrate that genetic risk for AD related to the *CLU* rs11136000 genotype is associated with higher EEG alpha2 desynchronization in frontal regions in clinically healthy subjects starting from 30 years of age. This indicates neurophysiological hyperactivation during the letter fluency task in the absence of any differences in cognitive performance. In *CLU CC* carriers, alpha2 ERD exhibited a significant correlation with age. The results suggest the systematic vulnerability of LFT-related cognitive networks in people carrying the AD risk variant of the *CLU* gene.

## Figures and Tables

**Table 1:** Demographic information, *APOE* genotype, and letter fluency test (LFT) performance of nondemented individuals aged 19 to 80 years with different *CLU* genotypes.

	N	Age	Gender m/f	*APOE E4*−/*APOE E4*+	LFT (Words)
*CLU CC*	34	42.7 ± 3.0	16/18	19/15	49.0 ± 2.4
*CLU CT&TT*	60	47.9 ± 2.1	12/38	40/20	46.4 ± 1.8
*p*-values		0.14	0.04	0.38	0.38

Data are presented as means and standard errors. *APOE E4*−, individuals with ε3/ε3 and ε2/ε3 genotypes; *APOE E4*+, individuals with ε3/ε4 and ε4/ε4 genotypes; m, men; w, women; LFT, letter fluency test.

**Table 2:** Demographic information, *APOE* genotype, and LFT performance of nondemented individuals aged 30 to 80 years with different *CLU* genotypes.

	N	Age	Gender m/f	*APOE E4*−/*APOE E4*+	LFT (Words)
*CLU CC*	24	50.6 ± 2.9	11/13	12/12	48.8 ± 3.4
*CLU CT&TT*	48	54.3 ± 1.5	18/30	31/17	45.1 ± 2.0
*p* values		0.22	0.61	0.31	0.33

Abbreviations are the same as in Table 1.

## Data Availability

Not applicable.

## Abbreviations

AD: Alzheimer’s disease; EOAD, early onset AD; LOAD, late-onset AD; QEEG, quantitative electroencephalography; ERD/S, event-related desynchronization/synchronization; MCI, mild cognitive impairment; GWAS, genome-wide association study; Chr, chromosome; *APOE*, apolipoprotein E; *CLU*, clusterin; *IL1TAP*, interleukin1 receptor accessory protein genotype; Neuron navigator 2, *NAV2* gene; MEG, magnetoencephalography; fMRI, functional magnetic resonance imaging; BOLD, blood oxygenation level-dependent; Aβ, amyloid β; CDR, Clinical Dementia Rating scale; RFLP, restriction fragment length polymorphism.

## References

[1] Alzheimer’s Association (2020). 2020 Alzheimer’s disease facts and figures. Alzheimers Dement..

[2] Gu J.L., Liu F. (2020). Tau in Alzheimer’s Disease: Pathological Alterations and an Attractive Therapeutic Target. Curr. Med. Sci..

[3] Kawas C.H., Legdeur N., Corrada M.M. (2021). What have we learned from cognition in the oldest-old. Curr. Opin. Neurol..

[4] Karlsson I.K., Escott-Price V., Gatz M., Hardy J., Pedersen N.L., Shoai M., Reynolds C.A. (2022). Measuring heritable contributions to Alzheimer’s disease: Polygenic risk score analysis with twins. Brain Commun..

[5] Goate A., Chartier-Harlin M.C., Mullan M., Brown J., Crawford F., Fidani L., Giuffra L., Haynes A., Irving N., James L. (1991). Segregation of a missense mutation in the amyloid precursor protein gene with familial Alzheimer’s disease. Nature.

[6] Levy-Lahad E., Wasco W., Poorkaj P., Romano D.M., Oshima J., Pettingell W.H., Yu C.E., Jondro P.D., Schmidt S.D., Wang K. (1995). Candidate gene for the chromosome 1 familial Alzheimer’s disease locus. Science.

[7] Rogaev E.I., Sherrington R., Rogaeva E.A., Levesque G., Ikeda M., Liang Y., Chi H., Lin C., Holman K., Tsuda T. (1995). Familial Alzheimer’s disease in kindreds with missense mutations in a gene on chromosome 1 related to the Alzheimer’s disease type 3 gene. Nature.

[8] Sherrington R., Rogaev E.I., Liang Y., Rogaeva E.A., Levesque G., Ikeda M., Chi H., Lin C., Li G., Holman K. (1995). Cloning of a gene bearing missense mutations in early-onset familial Alzheimer’s disease. Nature.

[9] Saunders A.M., Strittmatter W.J., Schmechel D., George-Hyslop P.H., Pericak-Vance M.A., Joo S.H., Rosi B.L., Gusella J.F., Crapper-MacLachlan D.R., Alberts M.J. (1993). Association of apolipoprotein E allele epsilon 4 with late-onset familial and sporadic Alzheimer’s disease. Neurology.

[10] Schmechel D.E., Saunders A.M., Strittmatter W.J., Crain B.J., Hulette C.M., Joo S.H., Pericak-Vance M.A., Goldgaber D., Roses A.D. (1993). Increased amyloid beta-peptide deposition in cerebral cortex as a consequence of apolipoprotein E genotype in late-onset Alzheimer disease. Proc. Natl. Acad. Sci. USA.

[11] Farrer L.A., Cupples L.A., Haines J.L., Hyman B., Kukull W.A., Mayeux R., Myers R.H., Pericak-Vance M.A., Risch N., van Duijn C.M. (1997). Effects of age, sex, and ethnicity on the association between apolipoprotein E genotype and Alzheimer disease. A meta-analysis. JAMA.

[12] Korovaĭtseva G.I., Shcherbatykh T.V., Selezneva N.V., Gavrilova S.I., Golimbet V.E., Voskresenskaia N.I., Rogaev E.I. (2001). Genetic association between the apolipoprotein E (ApoE) gene alleles and various forms of Alzheimer’s disease. Genetika.

[13] Rogaev E.I. (1999). Genetic factors and a polygenic model of Alzheimer’s disease. Genetika.

[14] Harold D., Abraham R., Hollingworth P., Sims R., Gerrish A., Hamshere M.L., Pahwa J.S., Moskvina V., Dowzell K., Williams A. (2009). Genome-wide association study identifies variants at CLU and PICALM associated with Alzheimer’s disease. Nat. Genet..

[15] Lambert J.C., Heath S., Even G., Campion D., Sleegers K., Hiltunen M., Combarros O., Zelenika D., Bullido M.J., Tavernier B. (2009). Genome-wide association study identifies variants at CLU and CR1 associated with Alzheimer’s disease. Nat. Genet..

[16] Golenkina S.A., Goltsov A.J., Kuznetsova I.L., Grigorenko A.P., Andreeva T.V., Reshetov D.A., Kunizheva S.S., Shagam L.I., Morozova I.I., Goldenkova-Pavlova I.V. (2010). Analysis of clusterin gene (CLU/APOJ) polymorphism in Alzheimer’s disease patients and in normal cohorts from Russian populations. Mol. Biol..

[17] Bettens K., Brouwers N., Engelborghs S., Lambert J.C., Rogaeva E., Vandenberghe R., Le Bastard N., Pasquier F., Vermeulen S., Van Dongen J. (2012). Both common variations and rare non-synonymous substitutions and small insertion/deletions in CLU are associated with increased Alzheimer risk. Mol. Neurodegener..

[18] Foster E.M., Dangla-Valls A., Lovestone S., Ribe E.M., Buckley N.J. (2019). Clusterin in Alzheimer’s Disease: Mechanisms, Genetics, and Lessons from Other Pathologies. Front. Neurosci..

[19] Cai R., Han J., Sun J., Huang R., Tian S., Shen Y., Dong X., Xia W., Wang S. (2016). Plasma Clusterin and the CLU Gene rs11136000 Variant Are Associated with Mild Cognitive Impairment in Type 2 Diabetic Patients. Front. Aging Neurosci..

[20] Carrasquillo M.M., Crook J.E., Pedraza O., Thomas C.S., Pankratz V.S., Allen M., Nguyen T., Malphrus K.G., Ma L., Bisceglio G.D. (2015). Late-onset Alzheimer risk variants in memory decline, incident mild cognitive impairment and Alzheimer disease. Neurobiol. Aging.

[21] Ling I.F., Bhongsatiern J., Simpson J.F., Fardo D.W., Estus S. (2012). Genetics of clusterin isoform expression and Alzheimer’s disease risk. PLoS ONE.

[22] Elias A.K., Wilson M.R., Carver J.A., Musgrave I.F. (2022). The Extracellular Molecular Chaperone Clusterin Inhibits Amyloid Fibril Formation and Suppresses Cytotoxicity Associated with Semen-Derived Enhancer of Virus Infection (SEVI). Cells.

[23] Yerbury J.J., Poon S., Meehan S., Thompson B., Kumita J.R., Dobson C.M., Wilson M.R. (2007). The extracellular chaperone clusterin influences amyloid formation and toxicity by interacting with prefibrillar structures. FASEB J. Off. Publ. Fed. Am. Soc. Exp. Biol..

[24] Dong H.K., Gim J.A., Yeo S.H., Kim H.S. (2017). Integrated late onset Alzheimer’s disease (LOAD) susceptibility genes: Cholesterol metabolism and trafficking perspectives. Gene.

[25] Wu Z.C., Yu J.T., Li Y., Tan L. (2012). Clusterin in Alzheimer’s disease. Adv. Clin. Chem..

[26] Dubois B., Feldman H.H., Jacova C., Hampel H., Molinuevo J.L., Blennow K., DeKosky S.T., Gauthier S., Selkoe D., Bateman R. (2014). Advancing research diagnostic criteria for Alzheimer’s disease: The IWG-2 criteria. Lancet Neurol..

[27] Babiloni C., Blinowska K., Bonanni L., Cichocki A., De Haan W., Del Percio C., Dubois B., Escudero J., Fernández A., Frisoni G. (2020). What electrophysiology tells us about Alzheimer’s disease: A window into the synchronization and connectivity of brain neurons. Neurobiol. Aging.

[28] Babiloni C. (2022). The Dark Side of Alzheimer’s Disease: Neglected Physiological Biomarkers of Brain Hyperexcitability and Abnormal Consciousness Level. J. Alzheimers Dis. JAD.

[29] Moretti D.V., Zanetti O., Binetti G., Frisoni G.B. (2012). Quantitative EEG Markers in Mild Cognitive Impairment: Degenerative versus Vascular Brain Impairment. Int. J. Alzheimers Dis..

[30] Dauwels J., Vialatte F., Musha T., Cichocki A. (2010). A comparative study of synchrony measures for the early diagnosis of Alzheimer’s disease based on EEG. NeuroImage.

[31] Van Straaten E.C., Scheltens P., Gouw A.A., Stam C.J. (2014). Eyes-closed task-free electroencephalography in clinical trials for Alzheimer’s disease: An emerging method based upon brain dynamics. Alzheimers Res. Ther..

[32] Hata M., Kazui H., Tanaka T., Ishii R., Canuet L., Pascual-Marqui R.D., Aoki Y., Kanemoto H., Yoshiyama K., Iwase M. (2016). Functional connectivity assessed by resting state EEG correlates with cognitive decline of Alzheimer’s disease—An eLORETA study. Clin. Neurophysiol..

[33] Jeong H.T., Youn Y.C., Sung H.H., Kim S.Y. (2021). Power Spectral Changes of Quantitative EEG in the Subjective Cognitive Decline: Comparison of Community Normal Control Groups. Neuropsychiatr. Dis. Treat..

[34] Patchitt J., Porffy L.A., Whomersley G., Szentgyorgyi T., Brett J., Mouchlianitis E., Mehta M.A., Nottage J.F., Shergill S.S. (2022). Alpha3/alpha2 power ratios relate to performance on a virtual reality shopping task in ageing adults. Front. Aging Neurosci..

[35] Van Beijsterveldt C.E., Molenaar P.C., de Geus E.J., Boomsma D.I. (1996). Heritability of human brain functioning as assessed by electroencephalography. Am. J. Hum. Genet..

[36] Prichep L.S., John E.R., Ferris S.H., Rausch L., Fang Z., Cancro R., Torossian C., Reisberg B. (2006). Prediction of longitudinal cognitive decline in normal elderly with subjective complaints using electrophysiological imaging. Neurobiol. Aging.

[37] Van der Hiele K., Bollen E.L., Vein A.A., Reijntjes R.H., Westendorp R.G., van Buchem M.A., Middelkoop H.A., van Dijk J.G. (2008). EEG markers of future cognitive performance in the elderly. J. Clin. Neurophysiol. Off. Publ. Am. Electroencephalogr. Soc..

[38] Jelic V., Julin P., Shigeta M., Nordberg A., Lannfelt L., Winblad B., Wahlund L.O. (1997). Apolipoprotein E epsilon4 allele decreases functional connectivity in Alzheimer’s disease as measured by EEG coherence. J. Neurol. Neurosurg. Psychiatry.

[39] Lehtovirta M., Partanen J., Könönen M., Hiltunen J., Helisalmi S., Hartikainen P., Soininen H. (2000). A longitudinal quantitative EEG study of Alzheimer’s disease: Relation to apolipoprotein E polymorphism. Dement. Geriatr. Cogn. Disord..

[40] Canuet L., Tellado I., Couceiro V., Fraile C., Fernandez-Novoa L., Ishii R., Takeda M., Cacabelos R. (2012). Resting-state network disruption and APOE genotype in Alzheimer’s disease: A lagged functional connectivity study. PLoS ONE.

[41] De Waal H., Stam C.J., de Haan W., van Straaten E.C., Blankenstein M.A., Scheltens P., van der Flier W.M. (2013). Alzheimer’s disease patients not carrying the apolipoprotein E ε4 allele show more severe slowing of oscillatory brain activity. Neurobiol. Aging..

[42] Maturana-Candelas A., Gómez C., Poza J., Rodríguez-González V., Pablo V.G., Lopes A.M., Pinto N., Hornero R. (2021). Influence of PICALM and CLU risk variants on beta EEG activity in Alzheimer’s disease patients. Sci. Rep..

[43] Babiloni C., Benussi L., Binetti G., Cassetta E., Dal Forno G., Del Percio C., Ferreri F., Ferri R., Frisoni G., Ghidoni R. (2006). Apolipoprotein E and alpha brain rhythms in mild cognitive impairment: A multicentric electroencephalogram study. Ann. Neurol..

[44] Smailovic U., Johansson C., Koenig T., Kåreholt I., Graff C., Jelic V. (2021). Decreased Global EEG Synchronization in Amyloid Positive Mild Cognitive Impairment and Alzheimer’s Disease Patients-Relationship to *APOE ε4*. Brain Sci..

[45] Gutiérrez-de Pablo V., Gómez C., Poza J., Maturana-Candelas A., Martins S., Gomes I., Lopes A.M., Pinto N., Hornero R. (2020). Relationship between the Presence of the *ApoE ε4* Allele and EEG Complexity along the Alzheimer’s Disease Continuum. Sensors.

[46] Ponomareva N.V., Korovaitseva G.I., Rogaev E.I. (2008). EEG alterations in non-demented individuals related to apolipoprotein E genotype and to risk of Alzheimer disease. Neurobiol. Aging..

[47] Lee T.W., Yu Y.W., Hong C.J., Tsai S.J., Wu H.C., Chen T.J. (2012). The influence of apolipoprotein E Epsilon4 polymorphism on qEEG profiles in healthy young females: A resting EEG study. Brain Topogr..

[48] Ponomareva N., Andreeva T., Protasova M., Shagam L., Malina D., Goltsov A., Fokin V., Mitrofanov A., Rogaev E. (2013). Age-dependent effect of Alzheimer’s risk variant of CLU on EEG alpha rhythm in non-demented adults. Front. Aging Neurosci..

[49] Ponomareva N.V., Andreeva T.V., Protasova M.S., Shagam L.I., Malina D.D., Goltsov A.Y., Fokin V.F., Illarioshkin S.N., Rogaev E.I. (2017). Quantitative EEG during normal aging: Association with the Alzheimer’s disease genetic risk variant in PICALM gene. Neurobiol. Aging..

[50] Ponomareva N.V., Andreeva T.V., Protasova M., Konovalov R.N., Krotenkova M.V., Kolesnikova E.P., Malina D.D., Kanavets E.V., Mitrofanov A.A., Fokin V.F. (2022). Genetic association of apolipoprotein E genotype with EEG alpha rhythm slowing and functional brain network alterations during normal aging. Front. Neurosci..

[51] Filbey F.M., Slack K.J., Sunderland T.P., Cohen R.M. (2006). Functional magnetic resonance imaging and magnetoencephalography differences associated with APOE epsilon4 in young healthy adults. Neuroreport.

[52] Pfurtscheller G., Lopes da Silva F.H. (1999). Event-related EEG/MEG synchronization and desynchronization: Basic principles. Clin. Neurophysiol..

[53] Klimesch W., Sauseng P., Hanslmayr S. (2007). EEG alpha oscillations: The inhibition-timing hypothesis. Brain Res. Rev..

[54] Bookheimer S.Y., Strojwas M.H., Cohen M.S., Saunders A.M., Pericak-Vance M.A., Mazziotta J.C., Small G.W. (2000). Patterns of brain activation in people at risk for Alzheimer’s disease. N. Engl. J. Med..

[55] Lancaster T.M., Baird A., Wolf C., Jackson M.C., Johnston S.J., Donev R., Thome J., Linden D.E. (2011). Neural hyperactivation in carriers of the Alzheimer’s risk variant on the clusterin gene. Eur. Neuropsychopharmacol..

[56] Singh K.D., Barnes G.R., Hillebrand A., Forde E.M., Williams A.L. (2002). Task-related changes in cortical synchronization are spatially coincident with the hemodynamic response. NeuroImage.

[57] Benton A.L., Hamsher K.D. (1989). Multilingual Aphasia Examination.

[58] Wagner S., Sebastian A., Lieb K., Tüscher O., Tadić A. (2014). A coordinate-based ALE functional MRI meta-analysis of brain activation during verbal fluency tasks in healthy control subjects. BMC Neurosci..

[59] Baldo J.V., Schwartz S., Wilkins D., Dronkers N.F. (2006). Role of frontal versus temporal cortex in verbal fluency as revealed by voxel-based lesion symptom mapping. J. Int. Neuropsychol. Soc..

[60] Palva S., Palva J.M. (2007). New vistas for alpha-frequency band oscillations. Trends Neurosci..

[61] Nenert R., Viswanathan S., Dubuc D.M., Visscher K.M. (2012). Modulations of ongoing alpha oscillations predict successful short-term visual memory encoding. Front. Hum. Neurosci..

[62] Lenartowicz A., Lu S., Rodriguez C., Lau E.P., Walshaw P.D., McCracken J.T., Cohen M.S., Loo S.K. (2016). Alpha desynchronization and fronto-parietal connectivity during spatial working memory encoding deficits in ADHD: A simultaneous EEG-fMRI study. NeuroImage Clin..

[63] Logothetis N.K., Pauls J., Augath M., Trinath T., Oeltermann A. (2001). Neurophysiological investigation of the basis of the fMRI signal. Nature.

[64] Lee J.H., Durand R., Gradinaru V., Zhang F., Goshen I., Kim D.S., Fenno L.E., Ramakrishnan C., Deisseroth K. (2010). Global and local fMRI signals driven by neurons defined optogenetically by type and wiring. Nature.

[65] Gevins A., Smith M.E., McEvoy L., Yu D. (1997). High-resolution EEG mapping of cortical activation related to working memory: Effects of task difficulty, type of processing, and practice. Cereb. Cortex.

[66] Paek E.J., Murray L.L., Newman S.D. (2020). Neural Correlates of Verb Fluency Performance in Cognitively Healthy Older Adults and Individuals with Dementia: A Pilot fMRI Study. Front. Aging Neurosci..

[67] Klimesch W. (2012). α-band oscillations, attention, and controlled access to stored information. Trends Cogn. Sci..

[68] Reiman E.M., Quiroz Y.T., Fleisher A.S., Chen K., Velez-Pardo C., Jimenez-Del-Rio M., Fagan A.M., Shah A.R., Alvarez S., Arbelaez A. (2012). Brain imaging and fluid biomarker analysis in young adults at genetic risk for autosomal dominant Alzheimer’s disease in the presenilin 1 E280A kindred: A case-control study. Lancet Neurol..

[69] Jack C.R., Wiste H.J., Therneau T.M., Weigand S.D., Knopman D.S., Mielke M.M., Lowe V.J., Vemuri P., Machulda M.M., Schwarz C.G. (2019). Associations of Amyloid, Tau, and Neurodegeneration Biomarker Profiles with Rates of Memory Decline Among Individuals Without Dementia. JAMA.

[70] Bell R.D., Sagare A.P., Friedman A.E., Bedi G.S., Holtzman D.M., Deane R., Zlokovic B.V. (2007). Transport pathways for clearance of human Alzheimer’s amyloid beta-peptide and apolipoproteins E and J in the mouse central nervous system. J. Cerebral Blood Flow Metab..

[71] Palop J.J., Mucke L. (2010). Amyloid-beta-induced neuronal dysfunction in Alzheimer’s disease: From synapses toward neural networks. Nat. Neurosci..

[72] Tenke C.E., Kayser J., Abraham K., Alvarenga J.E., Bruder G.E. (2015). Posterior EEG alpha at rest and during task performance: Comparison of current source density and field potential measures. Int. J. Psychophysiol..

[73] Koelewijn L., Lancaster T.M., Linden D., Dima D.C., Routley B.C., Magazzini L., Barawi K., Brindley L., Adams R., Tansey K.E. (2019). Oscillatory hyperactivity and hyperconnectivity in young *APOE*-ɛ4 carriers and hypoconnectivity in Alzheimer’s disease. eLife.

[74] Putcha D., Brickhouse M., O’Keefe K., Sullivan C., Rentz D., Marshall G., Dickerson B., Sperling R. (2010). Hippocampal hyperactivation associated with cortical thinning in Alzheimer’s disease signature regions in non-demented elderly adults. J. Neurosci..

[75] O’Brien J.L., O’Keefe K.M., LaViolette P.S., DeLuca A.N., Blacker D., Dickerson B.C., Sperling R.A. (2010). Longitudinal fMRI in elderly reveals loss of hippocampal activation with clinical decline. Neurology.

[76] Braskie M.N., Jahanshad N., Stein J.L., Barysheva M., McMahon K.L., de Zubicaray G.I., Martin N.G., Wright M.J., Ringman J.M., Toga A.W. (2011). Common Alzheimer’s disease risk variant within the CLU gene affects white matter microstructure in young adults. J. Neurosci..

[77] Roussotte F.F., Gutman B.A., Madsen S.K., Colby J.B., Thompson P.M., Alzheimer’s Disease Neuroimaging Initiative (2014). Combined effects of Alzheimer risk variants in the CLU and ApoE genes on ventricular expansion patterns in the elderly. J. Neurosci..

[78] Lancaster T.M., Brindley L.M., Tansey K.E., Sims R.C., Mantripragada K., Owen M.J., Williams J., Linden D.E. (2015). Alzheimer’s disease risk variant in CLU is associated with neural inefficiency in healthy individuals. Alzheimers Dement..

[79] Hughes C.P., Berg L., Danziger W.L., Coben L.A., Martin R.L. (1982). A new clinical scale for the staging of dementia. Br. J. Psych..

[80] Versavel M., Leonard J.P., Herrmann W.M. (1995). Standard operating procedure (SOP) for the registration and computer-supported evaluation of pharmaco-EEG data. Pharmacopsychiatry.

